# Ion Channels in The Pathogenesis of Endometriosis: A Cutting-Edge Point of View

**DOI:** 10.3390/ijms21031114

**Published:** 2020-02-07

**Authors:** Gaetano Riemma, Antonio Simone Laganà, Antonio Schiattarella, Simone Garzon, Luigi Cobellis, Raffaele Autiero, Federico Licciardi, Luigi Della Corte, Marco La Verde, Pasquale De Franciscis

**Affiliations:** 1 Department of Woman, Child and General and Specialized Surgery, University of Campania “Luigi Vanvitelli”, 80138 Naples, Italy; Gaetano.riemma7@gmail.com (G.R.); luigi.cobellis@unicampania.it (L.C.); raffaele.autiero@libero.it (R.A.); licciardi.federico@gmail.com (F.L.); marco.laverde88@gmail.com (M.L.V.); pasquale.defranciscis@unicampania.it (P.D.F.); 2 Department of Obstetrics and Gynecology, “Filippo Del Ponte” Hospital, University of Insubria, 21100 Varese, Italy; antoniosimone.lagana@uninsubria.it (A.S.L.); simone.garzon@univr.it (S.G.); 3 Department of Neuroscience, Reproductive Sciences and Dentistry, School of Medicine, University of Naples Federico II, 80131 Naples, Italy; dellacorte.luigi25@gmail.com

**Keywords:** endometriosis, ion channels, etiology, pathogenesis, CFTR, aquaporin, chloride channels

## Abstract

Background: Ion channels play a crucial role in many physiological processes. Several subtypes are expressed in the endometrium. Endometriosis is strictly correlated to estrogens and it is evident that expression and functionality of different ion channels are estrogen-dependent, fluctuating between the menstrual phases. However, their relationship with endometriosis is still unclear. Objective: To summarize the available literature data about the role of ion channels in the etiopathogenesis of endometriosis. Methods: A search on PubMed and Medline databases was performed from inception to November 2019. Results: Cystic fibrosis transmembrane conductance regulator (CFTR), transient receptor potentials (TRPs), aquaporins (AQPs), and chloride channel (ClC)-3 expression and activity were analyzed. CFTR expression changed during the menstrual phases and was enhanced in endometriosis samples; its overexpression promoted endometrial cell proliferation, migration, and invasion throughout nuclear factor kappa-light-chain-enhancer of activated B cells-urokinase plasminogen activator receptor (NFκB-uPAR) signaling pathway. No connection between TRPs and the pathogenesis of endometriosis was found. AQP5 activity was estrogen-increased and, through phosphatidylinositol-3-kinase and protein kinase B (PI3K/AKT), helped in vivo implantation of ectopic endometrium. In vitro, AQP9 participated in extracellular signal-regulated kinases/p38 mitogen-activated protein kinase (ERK/p38 MAPK) pathway and helped migration and invasion stimulating matrix metalloproteinase (MMP)2 and MMP9. ClC-3 was also overexpressed in ectopic endometrium and upregulated MMP9. Conclusion: Available evidence suggests a pivotal role of CFTR, AQPs, and ClC-3 in endometriosis etiopathogenesis. However, data obtained are not sufficient to establish a direct role of ion channels in the etiology of the disease. Further studies are needed to clarify this relationship.

## 1. Introduction

Endometriosis, defined as the presence of endometrial-like tissue outside the uterine cavity, is an estrogen-dependent benign disease that affects about 10% of reproductive-age women [1,2,3,4]. Of women affected by this pathology, 30%–50% suffer from pelvic pain and/or infertility [3,5,6,7]. Laparoscopy is considered the gold standard for the diagnosis and treatment of ectopic endometrial-like implants [3,8], however, 40% of treated women refer to a recurrence of the symptoms within five years [9,10,11,12], especially without post-operative pharmacological treatments [2,13,14]. Furthermore, endometriosis symptoms are often associated with a significant impairment in psychological wellbeing [15,16], that has also a substantial impact on the quality of life [17,18]. Several different theories have been developed in order to justify the etiopathology of endometriosis; although the theory of retrograde menstruation [19,20], developed by Sampson, was widely accepted several years ago, to date, accumulating evidence suggests a key role of genetics, epigenetics, and immune mechanisms for the onset and progression of the disease [21,22,23,24].

Ion channels are a heterogeneous group of transmembrane proteins that permit ions to flow across cell or organelle membranes [25,26,27]. When ions flow through channels, changes in membrane potential, intracellular pH, second-messenger pathways and intra-extracellular gradients are observed [28,29,30]. Those characteristics make ion channels crucial for the physiological homeostasis of neuronal signal transmission, as well as myofiber contraction, regulation of extra and intracellular volume, acid-base balance [31], and activation or inhibition of epithelial secretion [32]. At the same time, many pathways and physiological processes lead to strict regulation of their expression and functionality [26,33,34]. A wide spectrum of hormones, including progesterone, estradiol (E2), and growth factors, are known to act as modulators of the cellular expression of different ion channels [35,36]. Moreover, the open/close gating is dynamic; indeed, it can be switched by a variety of factors, including potential membrane changes, mechanical stimuli, temperature, and chemical substances, which allows ion channels to detect changes in the intracellular and extracellular environment and activate or deactivate secondary messengers for several signaling pathways [37,38]. Ion channels also play a significant role in balancing cell proliferation, apoptosis, and migration, which are fundamentally related to cancer development [39]. A significant number of different ion channels have been discovered in the endometrium, both in the epithelium and stroma of humans and animals [25]. Several studies have addressed the presence and altered function of ion channels in both eutopic and ectopic endometrium [40,41,42,43], suggesting that their overexpression may play an important role in the pathogenesis of endometriosis [1,44,45,46,47]. On that basis, our review aims to summarize these available pieces of evidence and discuss whether ion channels could be crucial in the migration and invasion of ectopic endometrial cells.

## 2. Materials and Methods

We performed a literature search on the MEDLINE database (accessed through PubMed) for articles written in English and published from inception to November 2019, in order to assess the search question “Does a relation between ion channels and etiopathogenesis of endometriosis exist?” The following Medical Subject Headings (MeSH) terms were used to screen and identify studies: “Endometriosis” (Unique ID: D004715), “ion channels” (Unique ID: D007473), “etiology” (Unique ID: Q000209).

Articles were excluded according to the following criteria: (a) articles were not written in English, (b) were published as conference papers or abstract only, and (c) studies including information that overlapped other publications. In the case of overlapping studies, we retrieved the most recent and/or most comprehensive manuscript. In our search, only articles concerning ion channels, with the exclusion of other genes or proteins, were included. The selection criteria for this narrative review included original articles (randomized and non-randomized clinical trials, including prospective observational studies, retrospective cohort studies, and case-control studies) and review articles regarding the potential role of ion channels on endometriosis development.

Articles that met the inclusion criteria were carefully read, and, when appropriate, further articles retrieved from their references were also reviewed in order to include other critical studies that might have been missed in the initial search. A total amount of eighty-eight references were thus used in this review. We presented here a narrative synthesis of the available evidence about the topic.

## 3. Cystic Fibrosis Transmembrane Conductance Regulator (CFTR) and Endometriosis

Cystic fibrosis transmembrane conductance regulator (CFTR) is a cyclic adenosine monophosphate (cAMP)-activated Cl^-^ and HCO_3_^--^ ion transporting channel, ubiquitously expressed in the epithelial cells of several tissues [48]. CFTR is essential in the regulation of epithelial fluid secretion, moving H_2_O into the organ lumen through a Cl^-^ efflux [49]. CFTR mutations cause cystic fibrosis, in which defective electrolyte and fluid transport can cause heterogeneous phenotypes of disease in different organs [48,50]. CTFR is expressed in the endometrial epithelium of guinea-pigs and other animals. In human endometrium, CFTR is also widely expressed, and its expression changes in a cyclic manner [43,51]. In cultured glandular cells, it was found to be upregulated by progesterone and downregulated by estradiol [51,52,53]. The expression and role of CFTR in endometriosis has been evaluated by Huang et al. [45]: in ectopic, endometrial-like samples, quantitative real-time polymerase chain reaction (qPCR) results demonstrated a significantly higher expression of CFTR mRNA and proteins in endometriotic lesions compared to normal endometria. Moreover, a CFTR signaling-mediated mechanism has been hypothesized to play a role in endometrial cell migration. Considering that CFTR-regulated cell migration was not dependent on its function as a channel, but by its interaction with other proteins, the aberrantly high levels of expression of CFTR might also be related to the high numbers of proteins that interact with this molecule.

The involvement of NFκB in acting as an intermediate for the effect of CFTR in endometrial cells, as well as the link between CFTR channel and NFκB, was already well described [48], although their direct relationship is still debated and controversial. Concerning cystic fibrosis, an inverse relationship between the two is well demonstrated; indeed, chronic inflammation of the lung, which is a key element of the disease, is strictly linked to the upregulation of NFκB that is found when CFTR is mutated [54]. In addition to this, the inverse relationship between NFκB and CFTR has also been found in the male reproductive tract, disrupting spermatogenesis in a similar way as in cryptorchidism. Moreover, a robust connection between CFTR and NFκB has also been found in the mouse embryo [55], and other female cancers (i.e. cervical cancer) [45]. Indeed, in human endometrial Ishikawa (ISK) cells, when overexpression of CFTR occurred, an enhanced cell migration with upregulated NFκB p65 and urokinase receptor (uPAR) pathway signaling was observed. Conversely, knockdown of CFTR was linked to inhibition of endometrial cell migration capacity. Furthermore, when curcumin or Bay were used to inhibit NFκB [55], they significantly reduced the expression of uPAR and overall cell migration in the CFTR-overexpressing ISK cells [45,53].

Huang et al. [45] also demonstrated the functional role of CFTR in endometrial cell migration. However, the CFTR-regulated cell migration ability was not correlated to its ion channel function but its expression level. These results suggest that CFTR does not directly act as an ion channel in the development of endometriosis: when a high aberrant expression of CFTR is reached, an abnormally high uPAR expression is achieved too; therefore, this may trigger the motility of endometrial cells, which is crucial for the progression of endometriosis [50].

## 4. Transient Receptor Potential (TRP) Channels and Endometriosis

Transient receptor potential (TRP) channels are known to be involved in the regulation of cell migration, adhesion, and proliferation, as well as neoangiogenesis [56]. The TRP superfamily consists of the following six subfamilies, which are based on sequence homology: ankyrin-rich (TRPA1), vanilloid (TRPV1-6), canonical (TRPC1-7), melastatin-like (TRPM1-8), polycystin (TRPP2/3/5), and mucolipin (TRPML1-3) [57]. Their localization is ubiquitary, and they can be activated by a wide number of molecules and stimuli [56,58,59]. In endometrial biopsies, TRP expression levels have been reported to be differently down- and upregulated during the different phases of the menstrual cycle [60]. High mRNA levels for TRPC1/4, TRPC6, TRPV2, TRPV4, TRPM4, and TRPM7 and the functional expression of TRPV2, TRPV4, TRPC6, and TRPM7 have been found in primary human endometrial stromal cells (hESC) [60]. Moreover, these channels were previously discovered to be somehow involved in several processes that regarded pathogenesis of endometriosis: TRPC1, TRPC4, and TRPV2 are involved in cell migration; TRPC4 in cell adhesion; and TRPM4, TRPM7, and TRPV2 have a crucial role in cell proliferation [47,61,62,63]. Persoons et al. [47] evaluated the expression profiles of TRP channels in endometrial biopsies from women with endometriosis taken at different times during the menstrual cycle. According to data analysis, several TRPs (TRPV1, TRPV2, TRPV4, TRPV6, TRPM4, TRPM6, TRPM7, TRPC1, TRPC3, TRPC4, and TRPC6) expression levels were higher than the detection limit. In addition, they reported that, for most of the TRP channels, mRNA levels were rising and falling according to different phases of the menstrual cycle. This difference was particularly significant for TRPM3 and TRPM6 between the follicular-late luteal phase and the early luteal phase of the menstrual cycle. Unlike CFTR channels, currently there is poor evidence about the regulation of TRPs by estrogens or progestogens. Nevertheless, it has been found that TRPV6 expression in ISK cells and normal endometrium could be upregulated by estrogen during the follicular phase [60,64]. In addition, TRPM2 mRNA expression is increased when an estrogen treatment is administered in human endometrium and hESC [64]. When estrogen and progesterone are both administered, TRPC1 mRNA has been found to increase. Furthermore, TRPC6 expression could be upregulated by estrogen in hESC [65]. TRPV2, TRPV4, TRPC1/4, and TRPC6 were expressed in hESC samples retrieved from women affected by endometriosis both at the molecular and functional levels. At the same time, the proliferation and migration assays were not affected by TRP expression, so this element raises further concerns and doubts regarding their role in the pathogenesis of the disease [40,47,60]. In addition, there were no significant differences between the RNA expression pattern of TRP channels comparing endometrial samples from eutopic and ectopic endometria. Although there might be no connection between TRPs and the etiopathogenesis of endometriosis, Bohonyi et al. [40] discovered that the expression levels of TRPA1 and TRPV1 were significantly different between DIE stroma and epithelium, as well as in DIE epithelium, when compared with control samples. Moreover, they found elevated stromal TRPV1 immunopositivity in DIE [40]. Interestingly, these findings correlated with dysmenorrhea and dyschezia severity; indeed, stromal and epithelial TRPA1 and TRPV1 immunoreactivities were directly correlated to the pain experienced by the patient. In synthesis, there might be no connection to the pathogenesis of endometriosis, despite the fact that the functional expression of several TRP channels has been found in the endometrium [47].

## 5. Aquaporins (AQPs) and Endometriosis

Aquaporins (AQPs) consist of a group of 25–34 kDa hydrophobic integral transmembrane channels [66]. These channels allow the physiologic rapid passive movement of H_2_O across the cell membrane in order to facilitate osmotic balance [66]. AQPs are ubiquitary across the human body, although they are based on a specific tissue-selective expression pattern [67]. Furthermore, their functions are of paramount importance in epithelial and endothelial cells, where their roles are clearly involved in fluid balance [68]. Besides their well-known peculiarities, it has been hypothesized that AQPs may be involved actively in cell migration, metabolism, and signal transduction [69]. AQP2, AQP5, AQP8, and AQP9 were usually found in endometrial samples [42,70]. Isoforms 2, 5, and 8 were mainly located in luminal and glandular epithelia, and positive immunostaining analysis of frequency was decreased in ectopic endometrium when compared with the eutopic one. Concerning the different expressions during menstrual phases, AQP2, 5, and 8 were found at a low-frequency rate in early-proliferative phase endometria but a higher frequency was observed in late proliferative and secretory phases [42]. In addition, Jiang et al. [46] found that AQP5 expression in hESC was increased by estradiol in a dose-dependent manner, because of an estrogen-responsive-element in the AQP5 promoter both in mice and in humans. Activating the phosphatidylinositol-3-kinase and protein kinase B (PI3K/AKT) pathway, isoform 5 could promote murine in vivo ectopic implants of endometrial-like cells due, at least in part, to pro-estrogenic enhancement [46]. Moreover, due to a low-frequency rate in late proliferative and secretory phases, AQP5 expression might be influenced by other factors, i.e., progesterone, as well [42,46]. In order to better investigate the role of AQ5 in endometriosis, Choi et al. [44] cultured hESC and transfected small interfering RNA (siRNA) of AQP1 to AQP9. They found that the expression for AQP2 and AQP8 was significantly higher than the other isoforms; moreover, the expression of AQP9 was decreased in the eutopic endometrium of patients with endometriosis when compared with the control group. In addition, when AQP9 was transfected throughout siRNA in hESCs, they found a significantly elevated expression of matrix metalloproteinases (MMPs) 2 and 9, which are essential for endometrial cell proliferation, migration, and invasiveness [71,72,73]. The MMP-9 gene is detected on chromosome 20q12-13 and is able to code an enzyme that directly participates in the degradation of collagen type IV and gelatin, which are the essential components of the basal membrane. Previous data suggest that the increased proteolytic activity, as well as the concomitant increase in the levels of the metalloproteinases, could be linked to the development of endometriosis. Moreover, a study by Chung et al. [74] found that MMP-9 plays a critical role in the implantation as well as invasion by ectopic endometrial tissue. The expression analysis of MMP-9 detected a significantly higher percentage of expression in ectopic endometrial tissues when compared with eutopic endometrial tissues [75]. Furthermore, MMP-9 was able to promote angiogenesis, which is argued to be a key process in the pathogenesis of endometriosis. Several coding single-nucleotide polymorphisms (SNPs) of MMP-9 were also identified, including MMP-9-1562C/T SNP. In addition to these findings, it has been recently demonstrated that the transcriptional activity of the −1562T allele was higher than the −1562C allele [76]. In synthesis, these polymorphisms may be able to alter the structure of MMP-9, giving the women an increased risk of developing endometriosis. The MMP2 gene can be found on chromosome 16q13-2. It encodes a critical enzyme for the reconstruction of the extracellular matrix (ECM) by targeting gelatin and type IV, V, VII, and X collagens. In order to demonstrate a role for MMP-2 in endometriosis, it has been found that women with endometriosis show increased MMP2 expression compared with healthy controls; meanwhile, the levels of the tissue inhibitors of metalloproteinase-2 (an inhibitor of MMP-2) mRNA were significantly lower. In agreement with these data, MMP-2 mRNA levels were found highly expressed in endometriosis tissues, especially in samples from patients with advanced disease [77]. In addition, Western Blot analysis reported increased expression of active (phosphorylated) extracellular signal-regulated kinases (ERK1/2) and phosphorylated p38 mitogen-activated protein kinase (MAPK). Taken together, these findings may suggest a role of AQP9 in the pathogenesis of endometriosis [44].

## 6. Chloride Channel-3 (ClC-3) and Endometriosis

Chloride channel-3 (ClC-3) is an ion channel encoded by the gene CLCN3. It belongs to the voltage-gated Cl^2^ channel superfamily [78]. It has critical roles regarding cellular electric activity and volume homeostasis, and it is also involved in cellular proliferation, migration, invasiveness, and apoptosis [79,80]. Since similar aspects between endometriosis and cancer are traceable, it has been hypothesized that the expression of ion channels like ClC-3 in endometriotic cells are at higher levels than in healthy cells, with an increased ability for migration and invasion. Indeed, chloride channels were found as crucial for the migration of human glioma cells [81] and the chloride channel-3 (ClC-3) chloride channel was directly involved in cancer cell migration and invasion from different types of cancer, suggesting that ClC-3 can be a key promoter of invasiveness [82,83,84]. Considering these elements, Guan et al. [85] investigated the role of ClC-3 in ectopic endometrial-like cells in order to evaluate their migration and invasion ability in women affected by endometriosis from an epigenetic perspective [86,87]. These authors found that ClC-3 expression was clearly upregulated in human endometriotic tissue samples. More intriguing, several studies have documented that the presence of chronic inflammation is a critical component of tumor development and progression, including endometriosis. Indeed, it is also well reported that ClC-3 plays a critical role in inflammation when upregulated [87]. Although the underlying mechanisms responsible for overexpression of ClC-3 in endometriosis remains unclear, the relationship between ClC-3 and chronic inflammation has been well elucidated over the last ten years. Several studies describe that ClC-3-dependent Cl^2^ efflux contributes to tumor necrosis factor (TNF)-α-induced cell inflammation and, therefore, leads to endothelial cell adhesion [88]. Guan et al. highlighted that the expression of the ClC-3 was significantly overexpressed at both mRNA and protein levels in ectopic lesions when compared with eutopic endometrial samples. At the same time, they suggested that the downregulation of ClC-3 expression was correlated to the inhibition of migration and invasion of hESCs [85]. Nonetheless, a strong positive correlation between ClC-3 and MMP9 was found; indeed, in ectopic hESCs, high levels of both proteins were found, and when ClC-3 was knocked down, MMP9 expression was significantly decreased [85]. Therefore, these findings may suggest that ClC-3, throughout the MMP9 upregulation, is involved in the pathogenesis of endometriotic lesions [85].

## 7. Conclusions

**Figure 1:**
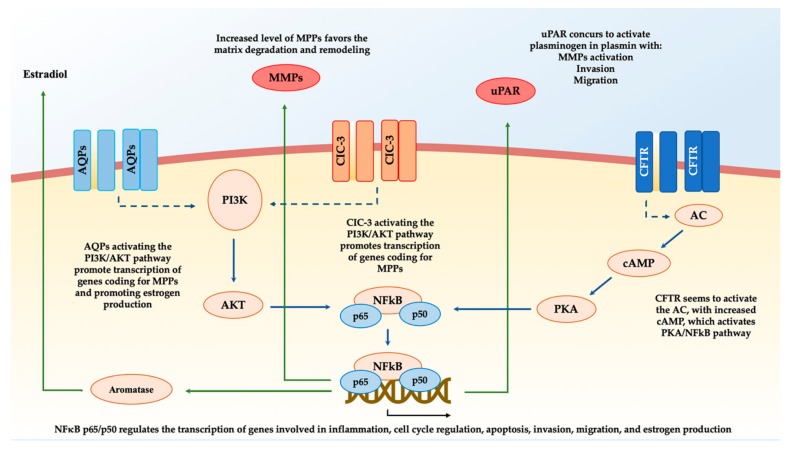
The main pathways involved in the pathogenesis of endometriosis mediated by ion channels. ClC-3: Chloride channel-3. AQPs: aquaporins. CFTR: cystic fibrosis transmembrane conductance regulator. AC: adenylate cyclase. cAMP: cyclical adenosine monophosphate. PKA: protein kinase A. PI3K: phosphatidylinositol-3-kinase. AKT: protein kinase B. MMPs: matrix metalloproteinases. uPAR: urokinase receptor.

The development of endometriosis is a process in which the endometrial stromal cells acquire and lose parts of their cellular function in order to gain the ability to proliferate, migrate, and invade outside the uterine cavity. Several keys factors characterize the pathogenesis and lead to heterogeneous phenotypes of the disease. In this process, different ion channels families are potentially related to the etiopathogenesis of endometriosis. CFTR, TRPs, AQPs, and ClC-3 expression and activity have been evaluated in both in vitro and in vivo experiments on ectopic and eutopic endometrium, hESCs, ISK cells, and murine models (for a summary of the findings of this review, refer to Table 1 and Figure 1).

CFTR expression was significantly higher in ectopic than eutopic endometrium and has been found to be regulated by estrogen and to fluctuate during the menstrual phases. Additionally, CFTR was able to upregulate the NFκB-p65-uPAR pathway, which orchestrates proliferation, migration, and invasiveness of endometrial cells. Several AQP isoforms were found related to the etiology of endometriosis; in particular, AQP5 was dose-dependently regulated by estrogens and able to activate the PI3K/AKT pathway, promoting implants of ectopic cells in vivo murine models. When AQP9 was down indeed regulated, the endometrial stromal cells’ migration and implantation index was enhanced by the upregulation of MMP2 and MMP9 throughout ERK/p38 MAPK signaling. ClC-3 achieved the same upregulation of MMP9 in hESCs, and, at the same time, a more significant cell migration and invasion activity were related to overexpression of ClC-3 in ectopic lesions. Taken together, these data suggest a potentially pivotal role of ion channels such as CFTR, AQPs, and ClC-3 in the complex and multifactorial pathogenesis of endometriosis. These families are able to activate several pathways and promote the capacity of endometrial cells to proliferate, migrate, and implant outside the uterus. Nevertheless, data available so far are still scarce and do not allow a firm conclusion about the topic to be drawn. For this reason, we take this opportunity to solicit further research to better understand the role of ion channels in the onset and progression of endometriosis and elucidate whether they might be considered potential targets for diagnosis and therapy.

## Figures and Tables

**Table 1:** The main ion channels involved in the pathogenesis of endometriosis.

Ion Channel	Regulation	Main Pathway	Action	References
CFTR	Upregulation	NFκB-p-65-uPAR	Migration; proliferation	[45]
AQP5	Upregulation	PI3K/AKT—MMP2, MMP9	Implantation	[46]
AQP9	Downregulation	ERK/p38 MAPK - MMP2, MMP9	Migration; implantation	[44]
ClC-3	Upregulation	MMP9	Implantation; inflammation	[85]

## Abbreviations

DIE	Deep infiltrating endometriosis
Wnt	Wingless-related integration site gene cluster
Hox	Homeobox genes
CFTR	Cystic fibrosis transmembrane conductance regulator
TRP	Transient receptor potential
AQP	Aquaporin
ClC	Chloride channel
NFκB	Nuclear factor kappa-light-chain-enhancer of activated B cells
uPAR	Urokinase plasminogen activator receptor
PI3K	Phosphatidylinositol-3-Kinase
AKT	Protein kinase B
ERK	Extracellular signal-regulated kinases
MAPK	Mitogen-activated protein kinases
MMP	Matrix metalloproteinase
MeSH	Medical subject headings
E2	Estradiol
qPCR	Quantitative real-time polymerase chain reaction
ISK	Human endometrial Ishikawa cell
hESC	Human endometrial stromal cell
siRNA	small interfering RNA
ECM	Extracellular matrix
SNP	Single-nucleotide polymorphism
TNF-α	Tumor necrosis factor α
